# Oct4 expression in gastric carcinoma: association with tumor proliferation, angiogenesis and survival

**DOI:** 10.1186/s43046-019-0005-0

**Published:** 2019-11-01

**Authors:** Dina M. El-Guindy, Rania E. Wasfy, Muhammad T. Abdel Ghafar, Dina A. Ali, Asmaa M. Elkady

**Affiliations:** 1 Pathology Department, Faculty of Medicine, Tanta University, Tanta, Egypt; 2 Clinical Pathology Department, Faculty of Medicine, Tanta University, Tanta, Egypt; 3 Clinical Oncology Department, Faculty of Medicine, Tanta University, Tanta, Egypt

**Keywords:** Octamer-binding transcription factor 4 (Oct4), Gastric carcinoma, Cancer stem cells (CSC)

## Abstract

**Background:**

Octamer-binding transcription factor 4 (Oct4) is a transcription factor that has an important role in stem cell differentiation and self-renewal. Oct4 has also been implicated in tumorigenicity of different cancers. This study aimed to analyze Oct4 expression in gastric carcinoma (GC) and to evaluate the relation between Oct4 expression and clinicopathologic parameters, tumor proliferation, and angiogenesis in addition to patient survival.

**Results:**

Oct4 mRNA was detected by quantitative reverse transcription PCR (qRT-PCR) in 45 GC specimens and adjacent non-cancerous tissues. We found a significant difference in Oct4 mRNA relative expression levels in GC tissue compared with adjacent non-cancerous tissues (*p* < 0.001). Furthermore, immunohistochemistry (IHC) was performed to study the Oct4 expression in GC cases. High Oct4 immunostaining was detected in 62.2% of GC specimens. High Oct4 expression both by mRNA relative quantitation and IHC were significantly related to poorly differentiated tumors, nodal metastasis, and stage III tumors. Moreover, high Oct4 IHC expression was also associated with cases positive for Ki-67 and VEGF expressions (*p* < 0.001 and 0.021, respectively). Oct4 expression identified by both mRNA relative quantitation and IHC was significantly related (*p* < 0.001). As regards patient survival, high Oct4 expression was significantly related to poor overall survival (OS) and disease-free survival (DFS) (*p* = 0.029 and 0.031, respectively).

**Conclusion:**

Oct4 plays a valuable role in the progression and prognosis of GC. High Oct4 expression is associated with high tumor grade, nodal metastasis, stage III tumors, and poor OS and DFS. High Oct4 is also significantly associated with Ki-67 and VGEF expression, thus enhancing tumor proliferation and angiogenesis.

## Background

Gastric carcinoma (GC) is one of the main causes of mortality-related cancers all over the world. For patients with surgically resectable GC, surgery with adjuvant chemo- and radiotherapy is the main way for treatment. However, many cases are still suffering from tumor recurrence and distant metastasis [1].

The theory of cancer stem cells (CSCs) focuses light on the cause and mechanism of recurrence after curative surgical resection and adjuvant therapy. CSCs, which have the ability for high self-renewal and proliferation, have been discovered also in so many solid cancers [2]. The relationship between various stem cell markers, clinicopathological characters of malignancies, and the prognostic value of these markers have been studied in GC. However, the results were still controversial [3].

Octamer-binding transcription factor 4 (Oct4) is a transcription factor that has a well-characterized value in stem cell differentiation and self-renewal. It is usually present in both embryonic and adult stem cells. It is also important for the maintenance of stem cell phenotypes and pluripotent characters [4]. Moreover, Oct4 plays an important role in the maintenance of tumor cell “stemness” [5]. Studies have shown overexpression of Oct4 in several somatic cancers such as oral squamous cell carcinoma, lung cancer, breast cancer, esophageal cancer, and gastric cancer. Ectopic Oct4 expression may be related to the progression of such cancers. Oct4 was also identified to be related to tumorigenesis and malignant transformation of tumors [6].

Tumor angiogenesis plays an important role in the proliferation, infiltration, and metastases of solid malignancies by promoting the delivery of oxygen, growth factors, and nutrients to tumor cells. Angiogenesis is regulated by specific essential factors. Vascular endothelial growth factor (VEGF) is considered one of the most important molecules promoting angiogenesis. The family of VEGF is composed of seven members: VEGF-A, VEGF-B, VEGF-C, VEGF-D, VEGF-E, VEGF-F, and placental growth factor. These molecules act through tyrosine kinase receptors (VEGF receptors), expressed mainly on endothelial cells [7].

This study aimed to investigate Oct4 expression in gastric cancer and to analyze the relation between Oct4 expression and clinicopathologic parameters, tumor proliferation, angiogenesis, and patient survival.

## Methods

### Study design

This study included 45 patients with non-distant metastatic pathologically proven gastric carcinoma. The study was carried out in the Pathology, Clinical Pathology, and Clinical Oncology Departments during the period between January 2015 and December 2016. Patients were followed up until December 2018.

### Patient characteristics and inclusion criteria

All included patients were free of distant metastases at the beginning of the study. Patients have age between 18 and 70 years, Karnofsky performance status ≥ 70, adequate bone marrow reserve (hemoglobin ≥ 10 g/dL, white blood cell count ≥ 3.5 × 10^9^/L, and platelets ≥ 100 × 10^9^/L), and good renal function (creatinine clearance ≥ 60 mL/min).

Patients were excluded from this study if they had metastases, altered mental status, dementia, or any psychiatric condition that affects understanding and impedes informed consent. Also, we excluded patients who had secondary malignancy or non-malignant systemic disease that precluded them from receiving chemotherapy (e.g., uncontrolled active infection, persistent immune-compromised states, congestive heart failure, any clinically significant cardiac arrhythmia). Patients who were pregnant and with clinically significant pleural effusions or ascites were also excluded from this study.

The protocol was approved by the Institutional Ethics Committee, and before the initiation of any treatment, all patients signed an informed consent.

### Treatment protocol and follow-up

All patients had undergone surgery with lymph node dissection and received more than four cycles of adjuvant chemotherapy. The regimen of chemotherapy was fluorouracil and/or cisplatin/oxaliplatin which consisted of 2000 mg/m^2^ (days 1 and 2) fluorouracil IV continuous infusion over 48 h and 50 mg/m^2^ (IV day 1) cisplatin, and this cycle was repeated every 14 days or oxaliplatin 85 mg/m^2^ (IV day 1), leucovorin 400 mg/m^2^ (IV day 1), fluorouracil 400 mg/m^2^ (IV push day 1), and fluorouracil 1200 mg/m^2^ (IV day 1, 2) continuous infusion over 24 h cycled every 14 days. Supportive care as growth factors, blood transfusions, and administration of antiemetics and analgesics were included, while prophylactic use of growth factors was not recommended.

The follow-up program consisted of physical examination and regular abdominal CT scan every 3–6 months for the first 2 years after operation. TNM stages were classified according to the American Joint Committee on Cancer (AJCC) [8].

### Tissue samples

From each participant in this study, gastric tissue specimens obtained by the surgical excision were sent to the Pathology Department for histopathological evaluation and immunohistochemical (IHC) staining. Samples from tumor center and adjacent non-cancerous tissue (at least 5 cm from the tumor) were then stored frozen at − 80 °C till genetically investigated [9].

### Histopathologic evaluation

Gastric carcinoma specimens were fixed in 10% neutral buffered formalin then paraffin blocks were prepared. Examination of hematoxylin and eosin (H&E)-stained sections was carried out to confirm the diagnosis of GC. Cases were histologically classified and graded according to the World Health Organization (WHO) [10].

### Immunohistochemical staining

Sections from GC tissue, on positively charged slides, were dried for 30 min at 37 °C. Deparaffinization and antigen retrieval were performed in a Dako PT Link unit. Both high and low pH EnVisionTM FLEX Target Retrieval Solutions were used reaching 97 °C for 20 min. Dako Autostainer Link 48 automated slide stainer was used for immunostaining. We used Oct4 mouse monoclonal antibody (clone MRQ-10, 1:30 dilution, Cell Marque, Rocklin, CA, USA), Ki-67 mouse monoclonal antibody (clone MIB-1, 1:100 dilution, Dako, Glostrup, Denmark), and VEGF mouse monoclonal antibody (M7273, 1:50 dilution, Dako, Glostrup, Denmark). Shortly, the slides were incubated with primary antibodies for 20–30 min following treatment with peroxidase-blocking reagent for 5 min then incubation with horseradish peroxidase (HRP) polymer reagent for 20 min and diaminobenzidine (DAB) chromogen/substrate working solution for 10 min. Hematoxylin was applied for counterstaining.

#### Evaluation of immunohistochemical staining

Oct4 expression was detected as a nuclear staining in gastric carcinoma cells. Oct4 was scored by multiplying the percentage of positive tumor cells and the staining intensity [11]. As regards Oct4 percentage, no positive tumor cells were graded 0; < 10% positive tumor cells, 1; 10–50% positive tumor cells, 2; and > 50% positive tumor cells, 3. Staining intensity was scored as follows: 0, no staining; 1, weak staining; 2, modest staining; and 3, strong staining. The final scores obtained were 0, 1, 2, 3, 4, 6, and 9. Tumors with scores ≤ 4 were considered low expression while scores ≥ 6 were regarded as high expression.

Positive Ki-67 expression was defined as brownish staining in the nuclei of 10% or more of tumor cells [12]. Cytoplasmic VEGF staining was regarded as positive when the percentage of stained tumor cell was 10 or more [13].

### Quantitative reverse transcription PCR

#### RNA extraction

The RNA was extracted from each stored frozen gastric tissue using RNA extraction kit (RNeasy mini kit, Qiagen, Hilden, Germany, Catalog no 74104) according to the manufacturer’s protocol. RNA yields were assayed quantitatively by measuring the absorbance at 260 nm on Jenway UV/Visible Spectrophotometer 6305, Staffordshire, UK.

#### Reverse transcription

The RNA yields were subjected to reverse transcription into cDNA using QuantiTect® Reverse Transcription (Qiagen, Hilden, Germany, Catalog no 205311), where the entire genomic DNA elimination reaction (14 μL) containing 500 ng of the template RNA mixed with 1 μL of Quantiscript Reverse Transcriptase, 4 μL of Quantiscript RT Buffer 5×, and 1 μL of RT primer mix, and incubated for 15 min at 42 °C then inactivated for 3 min at 95 °C according to the manufacturer’s instructions.

#### Relative quantitation Oct4 mRNA expression

RT-PCR amplifications with relative quantitation of Oct4 mRNA expression were performed using TaqMan gene expression assay kit (Thermo Scientific, Waltham, MA, USA). In a 20-μL total volume, mixture of 10.0 μL of 2× TaqMan® Universal PCR Master Mix II and 1.0 μL of 20× gene expression assay mix containing Oct4 primers (forward primer: 5-AGCAAAACCCGGAGGAGT-3; reverse primer: 5-CCACATCGGCCTGTGTATATC-3), with FAM-labeled probe (5-FAM-TGCAGGCCCGAAAGAGAAAGCG-3) and 5 μL of cDNA template (equivalent to 25 ng RNA), together with endogenous control (GAPDH) assay was used for each sample (forward primer: 5-ACCACAGTCCATGCCATCCAC-3; reverse primer: 5-TCCACCACCCTGTTGCTGTA-3). The plate was applied on Real-Time PCR System (Applied Biosystems, step I version) with the following thermal profile: hold at 95 °C for 10 min followed by 40 cycles (denaturation 95 °C for 15 s and annealing/extension at 60 °C for 1 min). The cycle threshold (CT) was obtained for the gene using Applied Biosystems, step I version, software analysis modules, and the expression of the gene was relatively quantified using the equation 2^−ΔΔCt^ [14].

### Statistical analysis

Statistical analysis was performed using Statistical Package for Social Science (SPSS version 23). Data were expressed as frequencies for categorical variables whereas continuous variables were expressed as mean ± SD or median and range. For comparing categorical variables, chi-square (*χ*^2^), Fisher’s exact, and Monte Carlo tests were applied. Continuous variables were compared using Student *t* test for normally distributed data, whereas Mann-Whitney and Kruskal-Wallis tests were performed for non-normally distributed ones. For survival analysis, overall survival (OS) rates were calculated as the interval between the date of diagnosis and the date of death or the last follow-up. Disease-free survival rates were calculated from the date of diagnosis to the date of disease recurrence and/or distant metastasis. Survival curves were built up using Kaplan-Meier method, and the exact log-rank test was used to evaluate the significance of the differences between the groups. *p* values of < 0.05 were considered statistically significant.

## Results

### Clinicopathological data

This study included 45 patients with gastric carcinoma. Twenty-eight were males while 17 were female with mean age of 60.76 ± 8.80 years. Most of the cases were located in the pylorus and fundus of the stomach [17 (37.8%) and 16 (35.6%), respectively]. Most of the cases have also a size more than or equal to 5 cm [30 cases (66.7%)]. Grossly, 23 cases (51.1%) had a solid appearance and 22 cases (48.9%) were with ulcerative configuration. On microscopic examination, the majority were of tubular type [32 cases (71.1%)]; majority of the cases were also in grade III [24 cases (53.3%)]. Regarding the stage of tumors, most of the cases had a lymph node metastasis [30 cases (66.7%)] and most of the cases were in the stage III group [21 cases (46.7%)]. Table 1 summarizes the clinicopathologic data of the studied cases.

**Table 1:** Clinicopathological characteristics of studied gastric carcinoma cases

Variable	Total, *N* (%)
Age (years) mean ± SD	60.76 ± 8.80
Gender	
Male	28 (62.2)
Female	17 (37.8)
Location	
Fundus	16 (35.6)
Body	12 (26.6)
Pylorus	17 (37.8)
Size	
< 5 cm	15 (33.3)
≥ 5 cm	30 (66.7)
Gross picture	
Solid	23 (51.1)
Ulcerative	22 (48.9)
Histologic type (WHO)	
Tubular	32 (71.1)
Mucinous	3 (6.7)
Signet ring	7 (15.5)
Papillary	3 (6.7)
Grade	
GI	8 (17.8)
GII	13 (28.9)
GIII	24 (53.3)
Nodal metastasis	
Negative	15 (33.3)
Positive	30 (66.7)
Staging	
I	6 (13.3)
II	18 (40)
III	21 (46.7)
Ki67	
Negative	19 (42.2)
Positive	26 (57.8)
VEGF	
Negative	14 (31.1)
Positive	31 (68.9)

*WHO* World Health Organization

### Relation between Oct4 immunohistochemical expression and clinicopathologic parameters

**Fig. 1:**
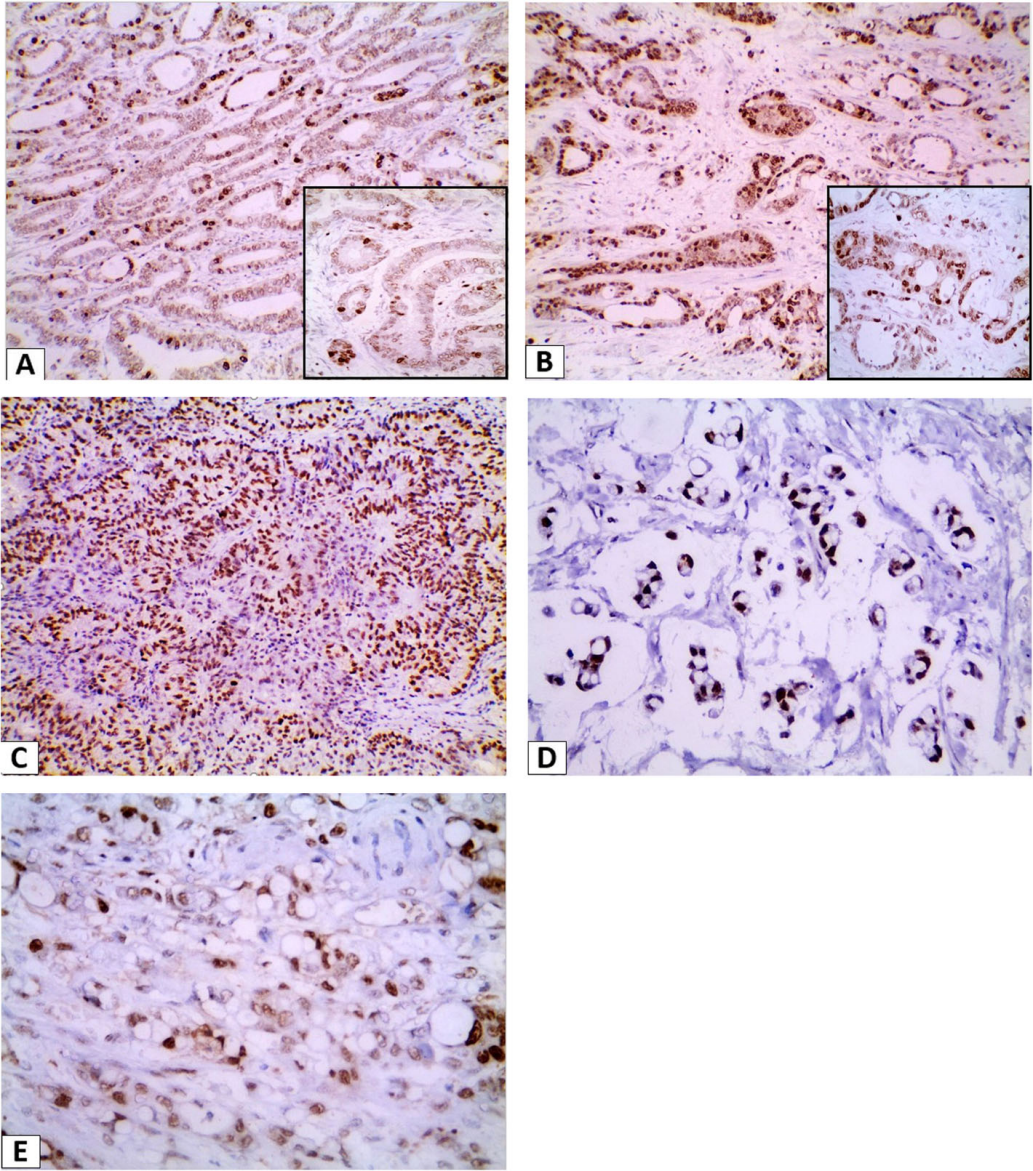
Oct4 immunohistochemical expression in gastric carcinoma cases: **a** Low Oct4 expression in well-differentiated gastric carcinoma (× 200) [inset: a higher magnification × 400]. **b** Low Oct4 expression in moderately differentiated gastric carcinoma (× 200) [inset: a higher magnification × 400]. **c** High Oct4 expression in poorly differentiated gastric carcinoma (× 200). **d** High Oct4 expression in mucinous carcinoma (× 400). **e** High Oct4 expression in signet ring carcinoma (× 400)

Among the 45 cases studied, Oct4 was highly represented in 28 cases (62.22%) while low expression was detected in the remaining 17 cases (37.78%). High Oct4 expression was significantly related to high tumor grade and stage III tumors. Most of grade III tumors [19 out of 24 (79.2%)] showed high Oct4 expression (*p* = 0.003). Sixteen out of 21 cases (76.2%) of stage III showed also high Oct4 expression (*p* = 0.031). Nodal metastasis was significantly associated with high Oct4 expression (*p* = 0.008). On the other hand, no significant associations were detected between Oct4 expression and age, gender, tumor location, size, gross appearance, and histologic types (Table 2, Fig. 1).

**Table 2:** Relation between Oct4 expression and clinicopathologic characteristics

	Total	Low Oct4 (*n* = 17), *N* (%)	High Oct4 (*n* = 28), *N* (%)	*p* value
Age in years (mean ± SD)		61.00 ± 10.71	60.61 ± 7.62	0.887
Gender				
Male	28	12 (42.9)	16 (57.1)	0.367
Female	17	5 (29.4)	12 (70.6)	
Location				
Fundus	16	6 (37.5)	10 (62.5)	0.511
Body	12	3 (25)	9 (75)	
Pylorus	17	8 (47.1)	9 (52.9)	
Size				
< 5 cm	15	3 (20)	12 (80)	0.110
≥ 5 cm	30	14 (46.7)	16 (53.3)	
Gross picture				
Solid	23	8 (34.8)	15 (65.2)	0.672
Ulcerative	22	9 (40.9)	13 (59.1)	
Histologic type (WHO)				
Tubular	32	12 (37.5)	20 (62.5)	0.873
Mucinous	3	1 (33.3)	2 (66.7)	
Signet ring	7	2 (28.6)	5 (71.4)	
Papillary	3	2 (66.7)	1 (33.3)	
Grade				
GI	8	7 (87.5)	1 (12.5)	0.003*
GII	13	5 (38.5)	8 (61.5)	
GIII	24	5 (20.8)	19 (79.2)	
Nodal metastasis				
Negative	15	10 (66.7)	5 (33.3)	0.008*
Positive	30	7 (23.3)	23 (76.7)	
Staging				
I	6	5 (83.3)	1 (16.7)	0.031*
II	18	7 (38.9)	11 (61.1)	
III	21	5 (23.8)	16 (76.2)	

*WHO* World Health Organization

*Statistically significant

### Evaluation of Oct4 mRNA relative expression

**Fig. 2:**
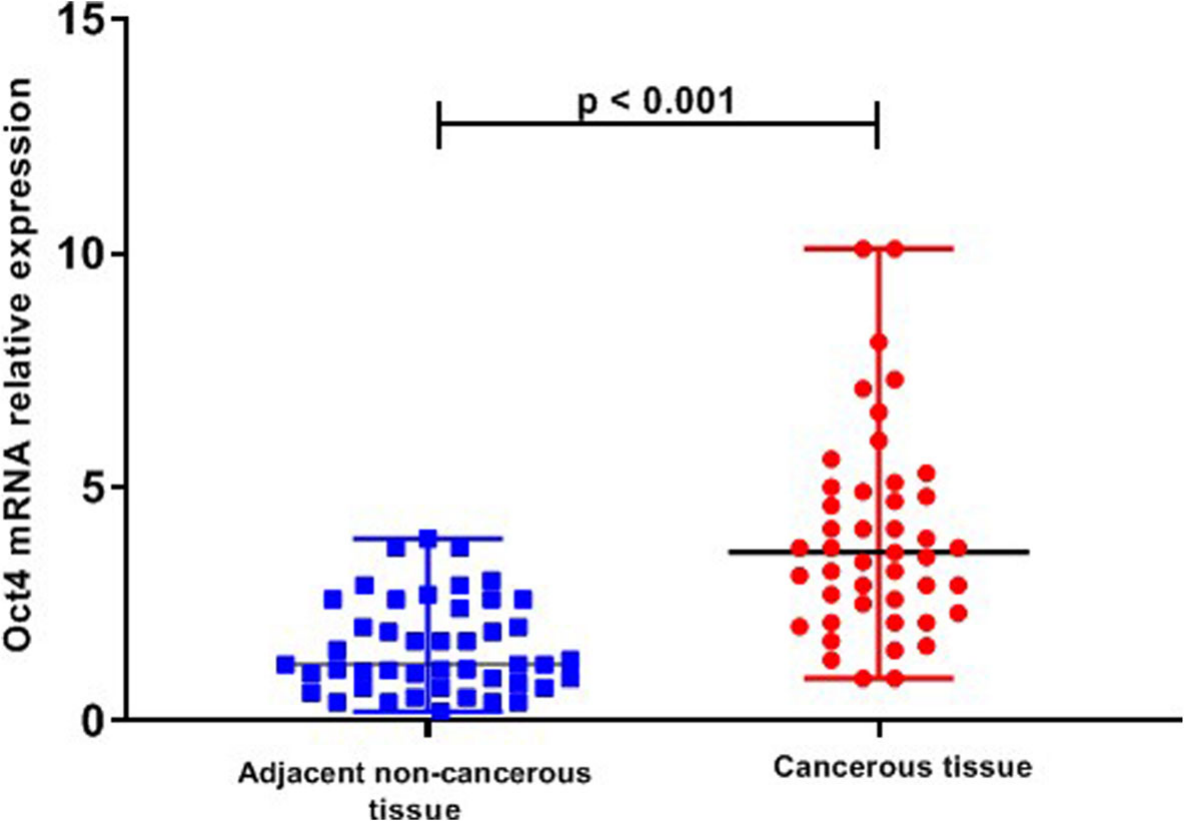
Oct4 mRNA relative expression in gastric carcinoma and adjacent non-cancerous tissues

Oct4 relative mRNA expression levels were found in all gastric cancerous and adjacent non-cancerous tissues of the entire included patients. In addition, statistically significant differences (*p* < 0.001) were detected in Oct4 mRNA relative expression levels in gastric cancerous tissue (median 3.6, range 0.9–10.1) compared with the adjacent non-cancerous tissues (median 1.2, range 0.2–3.9) as shown in Fig. 2.

**Fig. 3:**
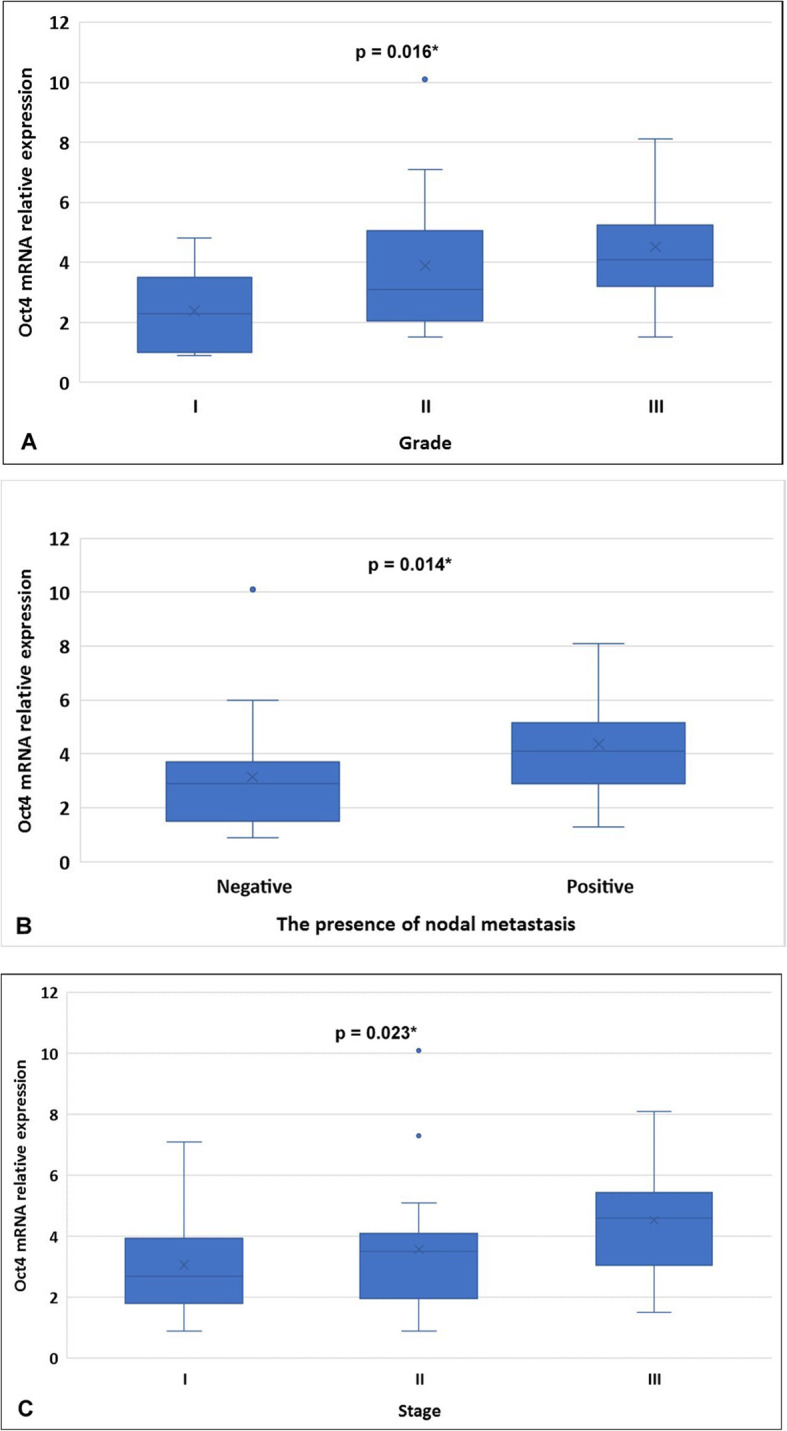
Relation of Oct4 mRNA relative expression levels and **a** tumor grade, **b** nodal metastasis, and **c** tumor stage

Higher Oct4 mRNA relative expression levels were significantly associated with grade III tumors (median 4.1, range 1.5–10.1), positive nodal metastasis (median 4.1, range 1.3–10.1), and stage III tumors (median 4.6, range 1.5–10.1) [*p* = 0.016, 0.014, and 0.023, respectively], as illustrated in Fig. 3.

### Relation between Oct4 expression and cell proliferation and angiogenesis

**Fig. 4:**
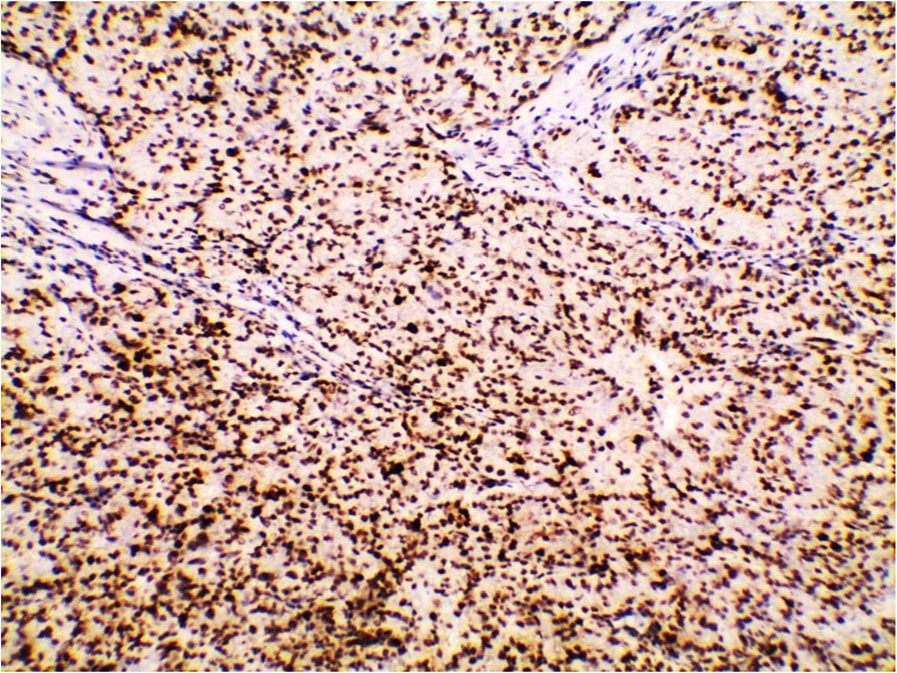
Positive nuclear Ki-67 expression in poorly differentiated gastric carcinoma (× 200)

**Fig. 5:**
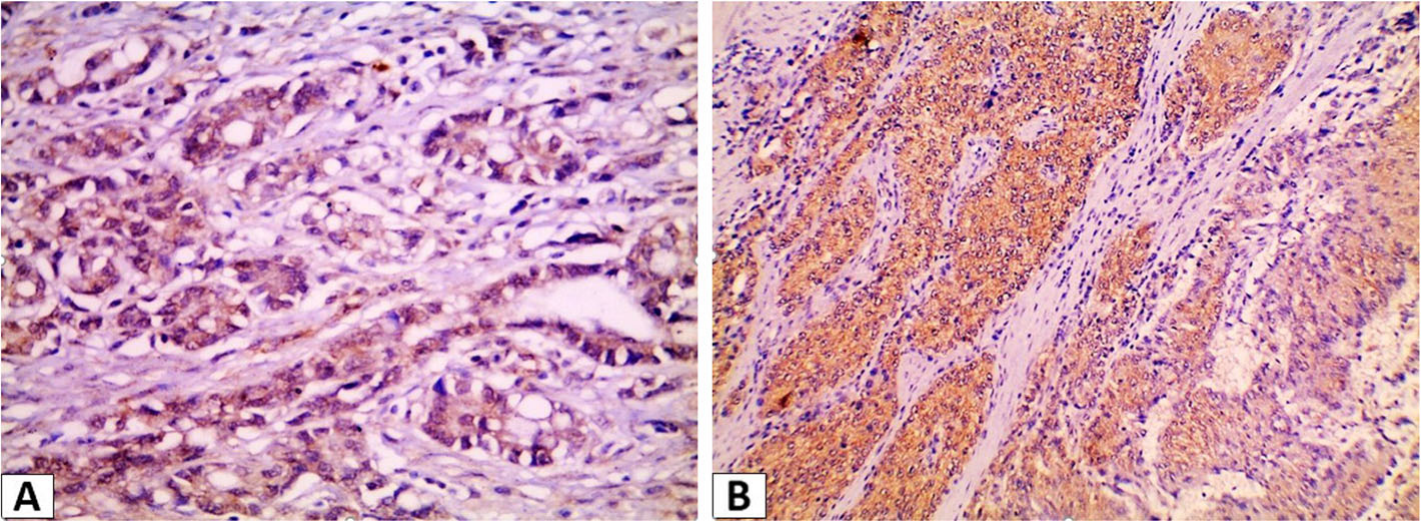
VEGF expression in gastric carcinoma cases. Positive VEGF cytoplasmic expression in **a** moderately differentiated carcinoma (× 400) and **b** poorly differentiated carcinoma (× 200)

As regards Oct4 IHC expression, high expression of Oct4 was significantly associated with positive Ki-67 nuclear staining in tumor specimens (*p* < 0.001). Twenty-two cases out of 26 representing 84.6% of Ki-67 positive tumors were associated with high Oct4 expression (Table 3, Fig. 4). A significant relation was also found between Oct4 expression and VEGF positivity (*p* = 0.021). Twenty-three out of 31 cases positive to VEGF showed high Oct4 expression (Table 3, Fig. 5).

**Table 3:** Relation between Oct4 expression with Ki-67 and VEGF

	Total	Low Oct4 (*n* = 17), *N* (%)	High Oct4 (*n* = 28), *N* (%)	*p* value
Ki 67				
Negative	19	13 (68.4)	6 (31.6)	< 0.001*
Positive	26	4 (15.4)	22 (84.6)	
VEGF				
Negative	14	9 (64.3)	5 (35.7)	0.021*
Positive	31	8 (25.8)	23 (74.2)	

*Statistically significant

A statistical significance difference in Oct4 mRNA relative expression levels was identified when comparing VEGF positive and negative tumors (*p* = 0.027), whereas no significant difference in Oct4 mRNA relative expression levels was detected among Ki-6 positive and negative tumors (*p* = 0.067).

### Relation between Oct4 mRNA relative expression and Oct4 immunohistochemical expression

**Fig. 6:**
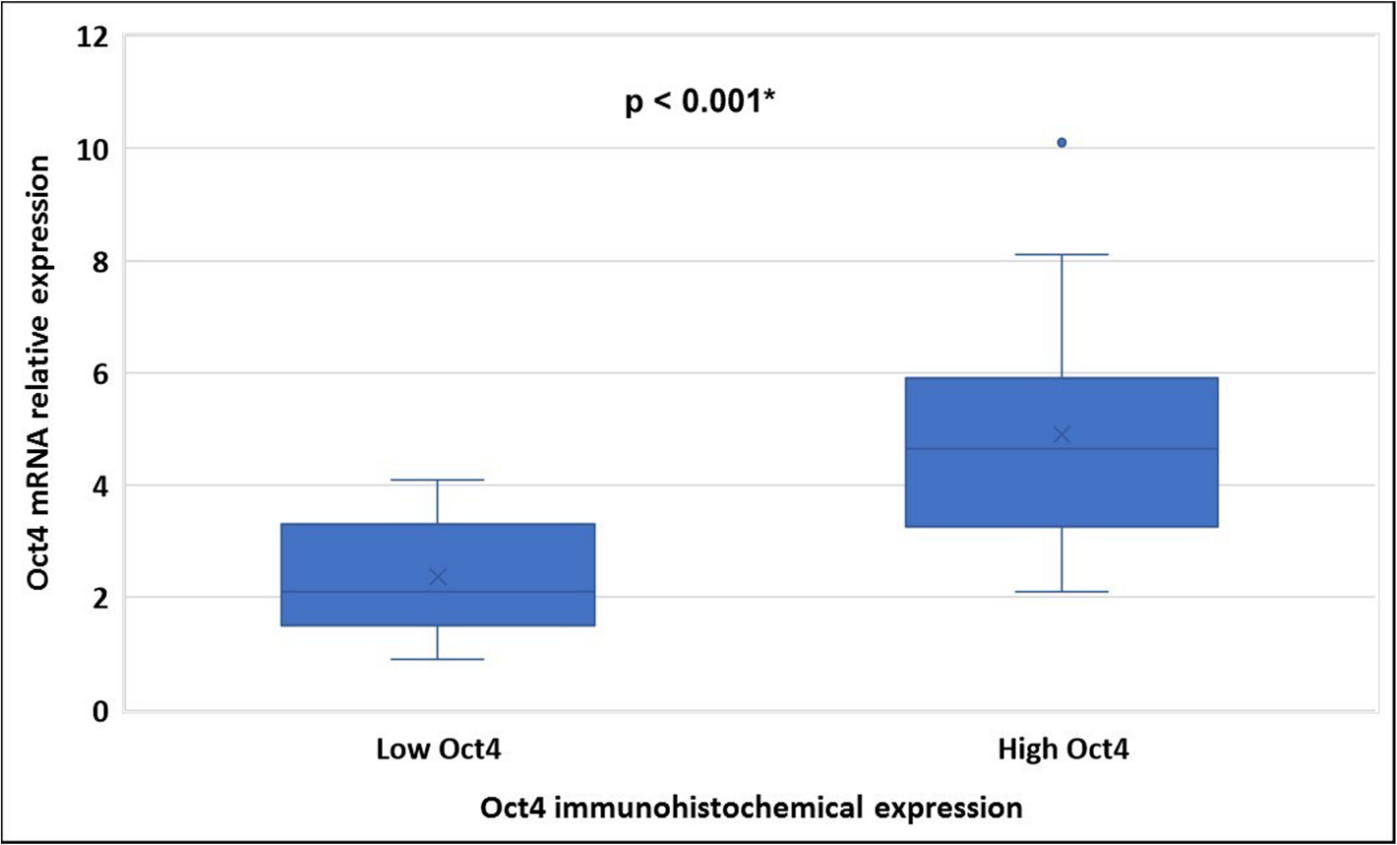
Relation between Oct4 mRNA relative expression and Oct4 immunohistochemical expression

Expression of Oct4 detected by mRNA relative quantitation and IHC was significantly related (*p* < 0.001). Oct4 mRNA relative expression levels (median 4.7, range 2.1–10.1) were significantly higher in tumors with high Oct4 immunohistochemical expression as shown in Fig. 6.

### Relation between Oct4 expression and patient survival

**Fig. 7:**
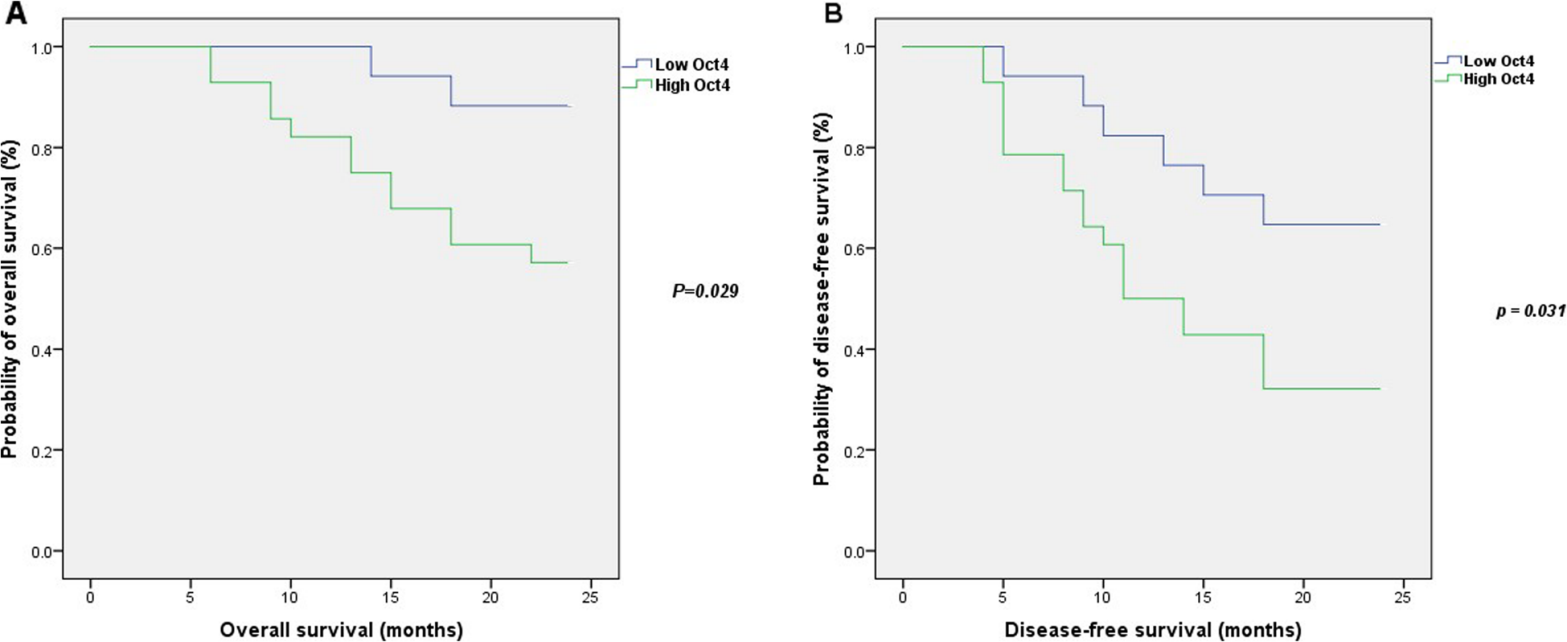
Kaplan-Meier curves for overall survival (OS) (**a**) and disease-free survival (DFS) (**b**) of gastric cancer patients with high or low Oct4 expression

The overall survival rate for those with high Oct4 expression was significantly lower than that in patients with low Oct4 expression. The 1- and 2-year survival rates were 100% and 88%, respectively, in the low-expression group, but only 85% and 57%, respectively, in the high-expression group (*p* value = 0.029, Fig. 7a). As regards disease-free survival, there was a significant difference between patients with high Oct4 expression and those with low expression (1- and 2-year DFS were 82.4% and 64.2% in the low-expression group versus 50% and 32.1% in the high-expression group (*p* value = 0.031, Fig. 7b).

## Discussion

Gastric carcinoma (GC) remains the third leading cause of cancer mortality worldwide. Patients with GC frequently develop cancer relapse and metastasis and are resistant to treatment. Therefore, it is of remarkable significance to investigate the mechanisms responsible for the poor prognosis in gastric cancer.

Over the past few years, growing evidences suggest that cancer stem cells (CSCs) have valuable roles in tumor aggressiveness, metastasis, chemotherapy resistance, and relapse [4]. Oct4 is an important transcriptional factor implicated in maintaining the pluripotency and self-renewal in CSCs; abnormal expression of Oct4 might contribute to carcinogenesis in different cancers [15].

To further investigate the relation between Oct4 expression and prognosis in gastric cancer, this study examined the association between Oct4 expression in GC and clinicopathologic parameters and patients’ survival. Also, relations between Oct4 expression and tumor proliferation and angiogenesis in GC were analyzed.

In the present study, we used qRT-PCR to investigate the Oct4 expression levels in tumoral and non-tumoral tissues. It was observed that the Oct4 expression level was highly elevated in GC tissues compared to the adjacent non-tumoral tissues. This agreed with Jiang et al. and Basati et al. [16, 17]. Moreover, Al-Marzoqee et al. found that Oct4 expression showed a significant increase from inflammation to dysplastic stage, and then malignant transformation, and thus concluded that Oct4 is implicated in the initial steps of gastric carcinogenesis [6].

Endogenous Oct4 knocking down or ectopic Oct4 overexpression are crucial in controlling the role that Oct4 plays in tumor initiation and propagation [18]. In a study by Beltran et al., they generated cell lines from Oct4 overexpression into normal breast cells. These cell lines were able to produce high-grade breast carcinoma in nude mice [19].

As regards Oct4 immunohistochemical expression in GC tissues, in this study, Oct4 was expressed as nuclear staining in 62.2% of the cases. The frequency of Oct4 expression as well as its subcellular localization varies among various studies. Li et al. observed Oct4 positivity as the main nuclear staining in 53.3% of the GC cases [20]. Jiang et al. reported positive Oct4 expression in 75.2% of GC specimens. Oct4 was predominantly cytoplasmic, with some nuclear localization [16]. In a study by Kong et al., Oct4 was detected in both the cytoplasm and the nucleus in 37.3% of the cases [5].

This wide variation in Oct4 expression may be due to the different antibodies used and different scoring system applied [15]. The variability in the Oct4 staining pattern may be due to the presence of isoforms for Oct4 generated by alternative splicing that displays different subcellular localization patterns [21].

The association between Oct4 expression in GC and clinicopathologic parameters remains controversial. The current work demonstrated that high Oct4 expression was significantly associated with high tumor grade, advanced stage, and nodal metastasis. Moreover, high Oct4 expression is significantly associated with poor overall survival (OS) than low Oct4 expression. Studies investigating the Oct4 expression in GC revealed similar results [3, 17, 20].

Similarly, Wang et al. and Rasti et al. reported an association between high Oct4 expression and poor prognosis and metastasis in hepatocellular and renal cell carcinomas, respectively [22, 23]. On the other hand, Matsuoka et al. suggested that Oct4 might repress the tumorigenic potential of GC cells. They reported that low Oct4 immunostaining significantly relates to invasive tumor, nodal metastasis, lymphatic invasion, and worse OS [24].

On analyzing the relation between the degree of Oct4 immunostaining and Ki67 expression. The present study reported a significant association between Oct4 immunostaining and Ki67 expression. Hu et al. reported that low Oct4 expression inhibits cell proliferation by promoting apoptosis in CSCs of lung cancer in vitro [25]. Also, Tsai et al. investigated the Oct4 expression in oral squamous cell carcinoma; they demonstrated that high oct4 expression is associated with increased tumor proliferation and enhanced invasive potential of cancer cells [26]. High expression of Oct4 could enhance tumorigenesis and metastasis by inducing cell proliferation, promoting tumor invasion, and inhibiting apoptosis through activating different genes and pathways [4].

Angiogenesis is necessary for maintaining tumor growth and metastasis. Little is known about the role of Oc4 in tumor angiogenesis. In this work, a significant relation was detected between Oct4 expression in GC and VEGF. Li et al. studied Oct4 expression in esophageal carcinoma; they found that Oct4 could activate epithelial-mesenchymal transition by means of increasing VEGF-C expression. This could enhance the invasive and metastatic potential of tumors [27]. In cervical carcinoma, Li et al. specified that isoform Oct4B could promote angiogenesis through the upregulation of CD34 and VEGF [21]. On the contrary, Chen et al. found no association between Oct4 expression and VEGF or microvessel density in non-small cell lung cancer [28].

An increasing number of studies have identified the essential role that hypoxia plays in regulating stem cell properties and functions including proliferative potential and differentiation. Covello et al. described that the expression of hypoxia-inducible factors (HIFs) is associated with overexpression of both Oct4 and VEGF [29]. Another study by Zhang et al. demonstrated that under hypoxic condition, the co-expression of Oct4 and HIF-2α develops and they act jointly to upregulate angiogenic factors including VEGF and promote angiogenesis [30].

Taken together, high Oct4 expression seems to be associated with tumor angiogenesis. But the exact mechanisms that regulate its association with VEGF need to be clarified in further studies.

## Conclusion

Oct4 plays a valuable role in the progression and prognosis of gastric carcinoma. High Oct4 expression is associated with high tumor grade, nodal metastasis, and stage III tumors. High Oct4 expression is significantly related to poor OS and DFS. High Oct4 is also significantly associated with Ki-67 and VGEF expression, thus enhancing tumor proliferation and angiogenesis.

## Data Availability

The datasets used and/or analyzed during the current study are available from the corresponding author on reasonable request.

## Abbreviations

AJCC: American Joint Committee on Cancer
CSCs: Cancer stem cells
DAB: Diaminobenzidine
DFS: Disease-free survival
GC: Gastric carcinoma
H&E: Hematoxylin and eosin
HIFs: Hypoxia-inducible factors
HRP: Horseradish peroxidase
IHC: Immunohistochemical
Oct4: Octamer-binding transcription factor 4
OS: Overall survival
qRT-PCR: Quantitative reverse transcription PCR
SD: Standard deviation
VEGF: Vascular endothelial growth factor

## References

[1] Sitarz R, Skierucha M, Mielko J, Offerhaus GJA, Maciejewski R, Polkowski WP. Gastric cancer: epidemiology, prevention, classification, and treatment. Cancer Manag Res. 2018;10:239–48. 29445300 10.2147/CMAR.S149619 PMC5808709

[2] Xu G, Shen J, Ou Yang X, Sasahara M, Su X. Cancer stem cells: the ‘heartbeat’ of gastric cancer. J Gastroenterol. 2013;48(7):781–97. 23184096 10.1007/s00535-012-0712-y

[3] Chen XL, Chen XZ, Wang YG, He D, Lu ZH, Liu K, et al. Clinical significance of putative markers of cancer stem cells in gastric cancer: a retrospective cohort study. Oncotarget. 2016;7(38):62049–69. 27557490 10.18632/oncotarget.11384 PMC5308710

[4] Fu Y, Du P, Zhao J, Hu C, Qin Y, Huang G. Gastric cancer stem cells: mechanisms and therapeutic approaches. Yonsei Med J. 2018;59(10):1150–8. 30450848 10.3349/ymj.2018.59.10.1150 PMC6240570

[5] Kong D, Su G, Zha L, Zhang H, Xiang J, Xu W, et al. Coexpression of HMGA2 and Oct4 predicts an unfavorable prognosis in human gastric cancer. Med Oncol. 2014;31(8):130–9. 25037576 10.1007/s12032-014-0130-5

[6] Al-Marzoqee FY, Khoder G, Al-Awadhi H, John R, Beg A, Vincze A, et al. Upregulation and inhibition of the nuclear translocation of Oct4 during multistep gastric carcinogenesis. Int J Oncol. 2012;41(5):1733–43. 22922943 10.3892/ijo.2012.1608

[7] Macedo F, Ladeira K, Longatto-Filho A, Martins SF. Gastric cancer and angiogenesis: is VEGF a useful biomarker to assess progression and remission? J Gastric Cancer. 2017;17(1):1–10. 28337358 10.5230/jgc.2017.17.e1 PMC5362829

[8] Ajani JA, In H, Sano T, Gaspar LE, Erasmus JJ, Tang LH, et al. Stomach In: Amin MB, Edge SB, Greene FL, et al., eds. AJCC cancer staging manual. 8th ed New York: Springer; 2017:203–220.

[9] Zhang J, Huang JY, Chen YN, Yuan F, Zhang H, Yan FH, et al. Whole genome and transcriptome sequencing of matched primary and peritoneal metastatic gastric carcinoma. Sci Rep. 2015;5:13750. 26330360 10.1038/srep13750 PMC4557136

[10] Lauwers GY, Carneiro F, Graham DY, Curado MP, Franceschi S, Montgomery E. Tumors of the stomach. In: Bosman FTCF, Hruban RH, Theise ND, editors. WHO Classification of Tumours of the Digestive System. 4th ed. Lyon: IARC Press; 2010. p. 48–58.

[11] Shen L, Huang X, Xie X, Su J, Yuan J, Chen X. High expression of SOX2 and OCT4 indicates radiation resistance and an independent negative prognosis in cervical squamous cell carcinoma. J Histochem Cytochem. 2014;62(7):499–509. 24710660 10.1369/0022155414532654 PMC4174620

[12] Sanaat Z, Halimi M, Ghojezadeh M, Pirovi AH, Gharamaleki JV, Ziae AEJE, et al. Immunohistochemical analysis of p53, Ki-67, CD44, HER-2/neu expression patterns in gastric cancer, and their association with one year survival in north-west of Iran. Int J Hematol-Oncol Stem Cell Res. 2013;7(3):15–20. 24505530 PMC3913147

[13] Zhang Z, Lin C, Chen S, Tu X, Wang L, Huang Q, et al. High tumor vascular endothelial growth factor expression is associated with poorer clinical outcomes in resected T3 gastric adenocarcinoma. Am J Clin Pathol. 2016;146(3):278–88. 27543975 10.1093/ajcp/aqw110

[14] Livak KJ, Schmittgen TD. Analysis of relative gene expression data using real-time quantitative PCR and the 2^−ΔΔCT^ method. Methods. 2001;25(4):402–8. 11846609 10.1006/meth.2001.1262

[15] Kim BW, Cho H, Choi CH, Ylaya K, Chung JY, Kim JH, et al. Clinical significance of OCT4 and SOX2 protein expression in cervical cancer. BMC Cancer. 2015;15:1015–22. 26706028 10.1186/s12885-015-2015-1 PMC4691290

[16] Jiang WL, Zhang PF, Li GF, Dong JH, Wang XS, Wang YY. Oct-4 is associated with gastric cancer progression and prognosis. Onco Targets Ther. 2016;9:517–22. 26869797 10.2147/OTT.S90031 PMC4734804

[17] Basati G, Mohammadpour H, Emami RA. Association of high expression levels of SOX2, NANOG, and OCT4 in gastric cancer tumor tissues with progression and poor prognosis. J Gastrointest Cancer. 2019:1–7. 10.1007/s12029-018-00200-x 30628031

[18] Wang YJ, Herlyn M. The emerging roles of Oct4 in tumor-initiating cells. Am J Physiol Cell Physiol. 2015;309(11):C709–18. 26447206 10.1152/ajpcell.00212.2015 PMC4725440

[19] Beltran AS, Rivenbark AG, Richardson BT, Yuan X, Quian H, Hunt JP, et al. Generation of tumor-initiating cells by exogenous delivery of OCT4 transcription factor. Breast Cancer Res. 2011;13(5):R94. 21952072 10.1186/bcr3019 PMC3262206

[20] Li N, Deng W, Ma J, Wei B, Guo K, Shen W, et al. Prognostic evaluation of Nanog, Oct4, Sox2, PCNA, Ki67 and E-cadherin expression in gastric cancer. Med Oncol. 2015;32(1):433–41. 25491144 10.1007/s12032-014-0433-6

[21] Li SW, Wu XL, Dong CL, Xie XY, Wu JF, Zhang X. The differential expression of OCT4 isoforms in cervical carcinoma. PLoS One. 2015;10(3):e0118033. 25816351 10.1371/journal.pone.0118033 PMC4376746

[22] Rasti A, Mehrazma M, Madjd Z, Abolhasani M, Saeednejad ZL, Asgari M. Co-expression of cancer stem cell markers OCT4 and NANOG predicts poor prognosis in renal cell carcinomas. Sci Rep. 2018;8(1):11739. 30082842 10.1038/s41598-018-30168-4 PMC6079110

[23] Wang G, Zhou H, Gu Z, Gao Q, Shen G. Oct4 promotes cancer cell proliferation and migration and leads to poor prognosis associated with the survivin/STAT3 pathway in hepatocellular carcinoma. Oncol Rep. 2018;40(2):979–87. 29901157 10.3892/or.2018.6491

[24] Matsuoka J, Yashiro M, Sakurai K, Kubo N, Tanaka H, Muguruma K, et al. Role of the stemness factors Sox2, Oct3/4, and Nanog in gastric carcinoma. J Surg Res. 2012;174(1):130–5. 21227461 10.1016/j.jss.2010.11.903

[25] Hu T, Liu S, Breiter DR, Wang F, Tang Y, Sun S. Octamer 4 small interfering RNA results in cancer stem cell–like cell apoptosis. Cancer Res. 2008;68(16):6533–40. 18701476 10.1158/0008-5472.CAN-07-6642

[26] Tsai LL, Hu FW, Lee SS, Yu CH, Yu CC, Chang YC. Oct4 mediates tumor initiating properties in oral squamous cell carcinomas through the regulation of epithelial-mesenchymal transition. PLoS One. 2014;9(1):e87207. 24475251 10.1371/journal.pone.0087207 PMC3903644

[27] Li C, Zhu M, Lou X, Liu C, Chen H, Lin X, et al. Transcriptional factor OCT4 promotes esophageal cancer metastasis by inducing epithelial-mesenchymal transition through VEGF-C/VEGFR-3 signaling pathway. Oncotarget. 2017;8(42):71933–45. 29069758 10.18632/oncotarget.18035 PMC5641101

[28] Chen Z, Wang T, Cai L, Su C, Zhong B, Lei Y, et al. Clinicopathological significance of non-small cell lung cancer with high prevalence of Oct-4 tumor cells. J Exp Clin Cancer Res. 2012;31(1):1–10. 22300949 10.1186/1756-9966-31-10 PMC3287152

[29] Covello KL, Kehler J, Yu H, Gordan JD, Arsham AM, Hu CJ, et al. HIF-2alpha regulates Oct-4: effects of hypoxia on stem cell function, embryonic development, and tumor growth. Genes Dev. 2006;20(5):557–70. 16510872 10.1101/gad.1399906 PMC1410808

[30] Zhang S, Zhao L, Wang J, Chen N, Yan J, Pan X. HIF-2α and Oct4 have synergistic effects on survival and myocardial repair of very small embryonic-like mesenchymal stem cells in infarcted hearts. Cell Death Dis. 2017;8:e2548–65. 28079892 10.1038/cddis.2016.480 PMC5386383

